# Fast and ballistic contractions involve greater neuromuscular power production in older adults during resistance exercise

**DOI:** 10.1007/s00421-022-04947-x

**Published:** 2022-04-16

**Authors:** Emmet J. Mc Dermott, Thomas G. Balshaw, Katherine Brooke-Wavell, Thomas M. Maden-Wilkinson, Jonathan P. Folland

**Affiliations:** 1 Versus Arthritis, Centre for Sport, Exercise and Osteoarthritis Research, Loughborough University, Leicestershire, UK; 2 School of Sport, Exercise and Health Sciences, Loughborough University, Leicestershire, LE11 3TU UK; 3 Physical Activity, Wellness and Public Health Research Group, Department of Sport and Physical Activity, Faculty of Health and Wellbeing, Collegiate Campus, Sheffield Hallam University, Sheffield, UK

**Keywords:** Resistance training prescription, Ageing, Neuromechanics, Muscle activation

## Abstract

**Purpose:**

Neuromuscular power is critical for healthy ageing. Conventional older adult resistance training (RT) guidelines typically recommend lifting slowly (2-s; CONV), whereas fast/explosive contractions performed either non-ballistically (FAST-NB) or ballistically (FAST-B, attempting to throw the load) may involve greater acute power production, and could ultimately provide a greater chronic power adaptation stimulus. To compare the neuromechanics (power, force, velocity, and muscle activation) of different types of concentric isoinertial RT contractions in older adults.

**Methods:**

Twelve active older adult males completed three sessions, each randomly assigned to one type of concentric contraction (CONV or FAST-NB or FAST-B). Each session involved lifting a range of loads (20–80%1RM) using an instrumented isoinertial leg press dynamometer that measured power, force, and velocity. Muscle activation was assessed with surface electromyography (sEMG).

**Results:**

Peak and mean power were markedly different, according to the concentric contraction explosive intent FAST-B > FAST-NB > CONV, with FAST-B producing substantially more power (+ 49 to 1172%, *P* ≤ 0.023), force (+ 10 to 136%, *P* < 0.05) and velocity (+ 55 to 483%, *P* ≤ 0.025) than CONV and FAST-NB contractions. Knee and hip extensor sEMG were typically higher during FAST-B than CON (all *P* < 0.02) and FAST-NB (≤ 50%1RM, *P* ≤ 0.001).

**Conclusions:**

FAST-B contractions produced markedly greater power, force, velocity and muscle activation across a range of loads than both CONV or FAST-NB and could provide a more potent RT stimulus for the chronic development of older adult power.

**Supplementary Information:**

The online version contains supplementary material available at 10.1007/s00421-022-04947-x.

## Introduction

Neuromuscular power is critical for healthy ageing, being the greatest functional predictor of mobility loss (Bassey et al. 1979), increased fall risk (Perry et al. 2007) and loss of physical independence (Foldvari et al. 2000) in older adults while also being the functional measure that declines most rapidly with ageing (i.e., declines more than strength; Skelton et al. 1994). Similarly, in osteoarthritis, one of the most common age-related musculoskeletal conditions, power has also been found to be a better predictor, than strength, of whole-body physical function (Accettura et al. 2015), self-reported function (Berger et al. 2012), knee joint mechanics (Murray et al. 2015), pain and quality of life (Reid et al. 2015). Therefore, maintaining/developing neuromuscular power is imperative for healthy ageing. Currently, older adults are recommended to engage in regular resistance training (RT) to improve and maintain physical function and health (ACSM guidelines, Ratamess et al. 2009; NSCA guidelines, Fragala et al. 2019; US government guidelines, Davies et al. 2019; UK government guidelines, Piercy et al. 2018). While there has been increasing attention and some recent recommendations for older adults to engage in RT for power development (light/moderate loads moved quickly; Fragala et al. 2019; Izquierdo et al. 2021), government recommendations for older adults emphasise conventional RT for strength development, i.e., relatively heavy loads moved slowly (US guidelines, Piercy et al. 2018; UK guidelines, Davies et al. 2019). One reason for the lack of consistent recommendations for the development of older adult power with RT is our limited knowledge of the neuromechanics (power, force, velocity, and neuromuscular activation) of RT in older adults and what types of contractions may be optimal for stimulating power development. Therefore, enhancing our knowledge of the acute neuromechanics of RT would seem like a key step prior to assessing the chronic adaptations to RT interventions in older adults.

Conventional RT (CONV) guidelines for the strength development of older adults typically prescribe contractions performed in a slow, controlled manner, i.e., a smooth sustained concentric lift over 2 s, with a high load (i.e., 60–80% of 1 repetition maximum [%1RM]). Conversely, RT recommendations for power development emphasise high velocity movements (concentric lift “as fast as possible”) and the use of lighter (i.e., 30–60%1RM) loads (Fragala et al. 2019; Kraemer et al. 2002). The neuromechanics of how these different methods of performing the lift (‘types of contractions’, i.e., conventional slow and controlled vs. fast/explosive) when lifting the same load remains unknown. Similarly, how this comparison may be affected by the load lifted (i.e., across the whole loading spectrum) is also unknown in older adults.

While RT for power development in older adults has begun to receive some scientific attention (Sayers and Gibson 2012; Balachandran et al. 2017; Rodriguez-Lopez et al. 2020), this has tended to focus on fast, but non-ballistic (FAST-NB) lifts; requirements which may be somewhat contradictory as the instruction/intention to move quickly is inevitably constrained by the requirement not to ballistically project/throw the load. In young adults FAST-NB contractions involve an extensive deceleration phase in the second half of the movement during which power production is negligible (Newton et al., 1996; Frost et al 2008), thus restricting the fast/explosive phase of the movement and likely the stimulus for power development. In contrast, fast ballistic (FAST-B) lifts where the intention is to move as fast as possible throughout the lift and ultimately project/throw the load as far as possible, may facilitate a wholly explosive movement with maximum velocity and power production throughout the concentric lift (Newton et al. 1996; Frost et al. 2008). Some support for this hypothesis is available from research in young, trained/athletic adults during upper body exercises, with FAST-B contractions found to generate greater peak and mean power, force and velocity compared to FAST-NB contractions. (Newton et al. 1996; Frost et al. 2008). However, even studies of young athletic adults report little consensus with no differences in power (Cronin and Marshall 2003) or force (Lake et al. 2012) in some comparisons of FAST-B and FAST-NB contractions. Critically, in older adults there is no information on the neuromechanics of FAST-B or FAST-NB contractions. Moreover, how any type of fast/explosive contractions (FAST-B or FAST-NB) compare to CONV contractions has not been examined in any population. Finally, given the importance of older adults maintaining/improving neuromuscular power of the lower body for mobility and well-being (Foldvari et al. 2000; Perry et al. 2007) the neuromechanics of lower body exercise would appear most relevant, but has had little attention in any population.

Measuring surface electromyography (sEMG) during contractions can help explain, in part, any mechanical differences found between different types of contractions. Therefore, if FAST-B contractions generate greater neuromuscular power, than FAST-NB or CONV contractions, this could be because of greater sEMG amplitude suggesting potentially greater levels of muscle activation. In trained young adults, FAST-B contractions have been found to illicit greater sEMG amplitude than FAST-NB contractions (Frost et al. 2008), but differences with CONV contractions, or for older adults, remain unexplored.

Therefore, the primary aim of this study was to compare the neuromuscular power produced during different types of RT contractions (conventional strength training, i.e., slow controlled, CONV vs. fast and non-ballistic, FAST-NB vs. fast and ballistic, FAST-B) in healthy older men performing concentric lifts with the full range of loads commonly used for RT (20, 35, 50, 65, and 80%1RM). Secondary aims included the assessment of the underlying determinants of power: force, velocity, sEMG amplitude as well as rate of force development. Mechanical variables were expressed as mean, peak and instantaneous values throughout the duration of the movement to comprehensively compare the different types of contractions. It was hypothesised that FAST-B would generate greater mean and peak power with all loads than CONV and FAST-NB, with FAST-NB constrained by the requirement not to ballistically project/throw the load leading to lower late phase muscle activation, force and velocity, and thus also power in comparison to FAST-B.

## Materials and methods

### Participants

Twelve older adult males (age, 67 ± 5 y; height, 1.80 ± 0.10 m; body mass, 73.5 ± 7.4 kg, BMI, 22.9 ± 2.2 kg·m^2^) volunteered to participate and provided written informed consent before completing this study that was approved by the Loughborough University Ethics Approval (Human Participants) Sub-Committee. Participants were recreationally active with a low to moderate level of mainly aerobic physical activity (2897 ± 1863 MET·min·week; e.g., walking, running, and cycling). Exclusion criteria were: no recent (previous 6 months) history of moderate or severe lower extremity musculoskeletal injury; no history of major surgery, musculoskeletal or neuromuscular disease in the involved leg; no medical conditions warranting exclusion from exercise and a BMI > 27 kg·m^2^. Participants were also excluded if they: scored < 23 on the mini-mental state exam (Folstein, et al., 1975), had blood pressure of > 150/90 mmHg (Reid et al. 2015) and took > 15 s to complete the sit-to-stand test (Buatois et al. 2010) Physical activity was assessed using the International Physical Activity Questionnaire (IPAQ short form, Craig et al., 2003).

### Experimental design

All participants visited the neuromuscular function laboratory on four separate occasions, consisting of one familiarisation session followed by three measurement sessions, 3–7 days apart. Each measurement session consisted of preliminary isometric maximum voluntary contractions (for normalisation of sEMG), before participants performed contractions with a range of progressively higher loads (20, 35, 50, 65 and 80%1RM) using one of three types of contractions (CONV or FAST-NB or FAST-B) for each measurement session, completed in a randomised order with 30 s rest between contractions and at least 2 min rest between loads. All measurement sessions were conducted at a consistent time of day for each participant, commenced between 12:00 and 19:00, and involved unilateral leg press contractions with an instrumented isoinertial leg press dynamometer for recording of force and displacement, that facilitated the derivation of velocity and power (see below). The dominant leg (*n* = 9) was assessed except when there was a history of dominant leg injury/surgery (*n* = 3; non-dominant leg). The familiarisation session involved preliminary measurement of one repetition maximum (1RM) for load prescription during the subsequent measurement sessions, practice of isometric maximum voluntary contractions (MVC), and practice efforts with all three types of contraction (4–5 efforts with each type of contraction, CONV followed by FAST-NB, then FAST-B) at each of two loads, 35 and 65% 1RM.

### Kinetic, kinematic and sEMG recordings

Participants were seated on the leg press dynamometer, with a fixed seat and adjustable (40 mm canvas webbing) straps used to restrain the pelvis and prevent any extraneous movement of the pelvis and torso during contractions. The dynamometer enabled measurements during a leg press action (simultaneous hip extension, knee extension and plantar flexion), with the participant ‘pressing’ against a plate loaded sled on a linear low friction track (30º inclined, see Supplemental Digital Copy 1). The sled was instrumented with a bespoke calibrated force plate consisting of four single axis load cells (CDC, model SP 3949; each 2 kN capacity; total capacity = 8 kN; Force Logic, Swallowfield, UK) in parallel in a rectangle formation (load cell spacing: length [0.25] x width [0.14 m]) secured between two aluminium plates, which was attached to the original foot plate of the sled and perpendicular to the sled track (see Supplemental Digital Content, 1B). Following extensive pilot work, to reduce ankle dorsiflexion and associated discomfort at the start of the leg press movement (common in older adults) a further modification involved mounting a rigid aluminium wedge on the force plate to provide a new surface foot plate at an angle of 21° to the force plate (surface area, 0.36 × 0.23 m). The leg-press dynamometer was constructed with multiple one-way adjustable mechanical catches, that effectively ‘caught’ the loaded sled once projected to facilitate safe projection of the sled during ballistic contractions.

For all contractions, the participants foot position was standardised/replicated in a central position on the surface foot plate using tape markers. For isometric MVCs and passive limb weight measurements only, the participant's foot was secured to the surface foot plate using a bespoke foot brace and adjustable strapping, that facilitated no active force and, therefore, a relaxed rested state during the passive measurements (see Supplemental Digital Content 1). A calibrated draw-wire transducer (WDS-2500-P96-SR-U, Micro-epsilon Ltd, Ortenburg, Germany) was used to assess displacement of the sled, with the spindle housing bolted to the static frame of the dynamometer, and the draw-wire attached to, and parallel to the movement of the sled (see Supplemental Digital Content 1A). The analogue force and displacement signals were sampled at 2,000 Hz using an external analogue to digital (A/D) converter (1401 Power 3, CED Ltd., Cambridge, UK), and recorded using Spike 2 computer software (CED Ltd., Cambridge, UK) on a personal computer.

Following the palpation and marking of the muscle borders and electrode positions, the skin was prepared by shaving, abrading and cleansing (70% ethanol). sEMG was recorded using a wireless EMG system (Trigno; Delsys Inc, Boston, MA, USA) with single differential Trigno sensors (inter-electrode distance = 1 cm) attached to the skin using an adhesive interface. sEMG sensors were positioned parallel to the presumed orientation of the muscle fibres. Two separate sEMG sensors were placed on each of the superficial quadriceps muscles (rectus femoris [RF], vastus lateralis [VL], vastus medialis [VM]) and positioned relative (%) to thigh length (greater trochanter to knee-joint space) measured from the superior aspect of the patella: RF_PROXIMAL_ (65%) and RF_DISTAL_ (55%), VL_PROXIMAL_ (55%) and VL_DISTAL_ (50%), VM_PROXIMAL_ (35%) and VM_DISTAL_ (30%). Single sEMG sensors were placed on each of the following superficial muscles: hamstrings (bicep femoris [BF], medial hamstring [MH]; 45% of thigh length measured from the popliteal fossa), gastrocnemius (lateral gastrocnemius [LG] and medial gastrocnemius [MG]; 75% and 85% of shank length (lateral malleolus to knee-joint space) measured from the calcaneus, respectively), soleus (SO; 66% shank length measured from the medial femoral condyle) and the gluteus maximus [GM; 50% of the distance between the second sacral vertebrae and the greater trochanter]. The raw sEMG signal was amplified at source (× 300; 20–450 Hz) before further amplification (overall total amplification =  × 909). The sEMG signal was sampled at 2000 Hz via the same A/D convertor as the force and displacement signals. To account for the inherent 48 ms delay present in the Delsys Trigno system, signals were time aligned during offline analysis.

## Familiarisation session and preliminary measurements

Familiarisation sessions first involved preliminary measurements of leg length, followed by participant’s practicing the isometric maximum voluntary contractions according to an identical protocol as for the measurement sessions (see below), that was followed by preliminary measurements of passive limb weight, and then one repetition maximum (1RM) lifting strength, in this order.

### Leg-length and passive limb weight

During familiarisation an individual reference position of full leg length (100% of leg length) was determined using the draw-wire displacement transducer with the knee extended and the leg relaxed and parallel to the sled track, with the plantar surface of the foot flat and central on the force plate. This reference position was used to prescribe individualised measurement positions and for normalisation of measurements throughout the range of motion during the isoinertial contractions, i.e., to percentage of leg length (%LL). The sled was fixed (i.e., stationary) in four different positions (95, 88, 81 and 74%LL). The passive limb weight exerted by the leg on the force plate (i.e., when relaxed and not contracting) was recorded and plotted against displacement to generate a quadratic function. This facilitated the interpolation of passive force at all positions throughout the range of motion for gravity correction during isoinertial contractions.

### One repetition maximum

During familiarisation, each participant’s leg press 1RM was determined and used for load prescription during the main measurement sessions. Participants performed preliminary lifts: two at light loads (~ 10–20 kg in addition to the mass of the sled, i.e., 29 kg), and a single lift with a moderate load (1.3 × body mass; ~ 80%1RM), with ~ 30 s rest between contractions. Thereafter, a series of near maximum lifts was undertaken to establish 1RM, with the load increased by ~ 2.5–5 kg after each successful lift. Each lift began in a stationary position at 74%LL and a successful lift was defined by the participant’s ability to move the load through the minimum specified displacement (74–95%LL). After each concentric lift, the load was lowered to the start position (74%LL) by the researchers (i.e., one either side of the leg press). 1RM was defined as the highest load that could be lifted through the specified displacement range, usually determined within 4–6 attempts, with each maximal attempt interspersed with ≥ 2 min of recovery.

### Measurement session

All measurement sessions were performed in the following order.

### Isometric maximum voluntary contractions

Isometric MVCs were performed to generate reference sEMG values for normalisation of sEMG during the isoinertial contractions. Participants performed a standardised isometric warm-up at one position (95%LL: 3 × 50%, 3 × 75% and 1 × 90% of perceived maximum force), with each contraction lasting ~ 3–5 s. Participants then performed 3–4 MVCs at each of two positions (95 then 81%LL). During each MVC participants were instructed to push as hard as possible for ~ 3–5 s, with 30 s rest between contractions and 2 min rest between positions. Biofeedback was provided with the force–time recording displayed prominently in front of the participant and a cursor used to indicate the highest force achieved during that series of MVCs.

During offline analysis, sEMG amplitude was assessed as root mean square (RMS) during a 500 ms epoch (250 ms either side) of isometric maximal voluntary force (iMVF, the highest single instantaneous force at each position). The RMS amplitude of each signal (recording site) was then baseline corrected (i.e., resting RMS amplitude was subtracted). The isometric position that produced the highest RMS amplitude for each functional muscle group (KE_EMG_, 81%LL; HE_EMG_ & PF_EMG_, 95%LL) was used for normalisation of that muscle group during the isoinertial contractions.

### Isoinertial contraction protocol and analysis

Participants performed 4–5 maximum effort contractions at each of five loads (ascending order: 20, 35, 50, 65 and 80%1RM), with 30 s rest between contractions and loads separated by ≥ 2 min, using either conventional (CONV), fast non ballistic (FAST-NB) or fast ballistic (FAST-B) contractions during each measurement session, in a randomised order. For CONV contractions participants were instructed to perform the concentric phase of the lift over 2 s, with participants receiving audio feedback of the lifting duration using a metronome. Finally, participants were instructed to maintain contact with foot plate throughout the lift (i.e., no ballistic projection of the load). For FAST-NB contractions participants were instructed to push “as fast as possible” initially during the concentric lifting portion, but to decelerate sufficiently during the latter part of the lift so as to maintain contact with force plate throughout the lift (i.e., no ballistic projection of the load). Finally, FAST-B contractions involved participants performing the concentric portion “as fast as possible” throughout the entire concentric lifting phase, with the load thrown/projected as far as possible.

During offline analysis, force and displacement signals were filtered using a low-pass second-order zero-lag (both directions) Butterworth filter with a cutoff frequency of 30 Hz. The filtered displacement signal was used to derive velocity (time constant = 15 ms). Force data were gravity corrected by subtracting passive force due to the passive weight of the limb measured statically (see above) to derive active force as the criterion measure of force. Instantaneous external contractile power was calculated as the product of active force and velocity measurements (P = F x V). The two contractions with highest instantaneous peak power for each load and type of contraction were analysed in detail and measurements were averaged. Mean power, force and velocity were averaged throughout the concentric movement duration (i.e., lifting motion) measured from displacement onset to force offset. The displacement onset was defined as the point the displacement signal increased above the baseline noise envelope during the 300 ms prior to displacement onset and did not return. The end of the concentric phase of contraction was defined as: force offset (active force = 0) when the load was projected/thrown (i.e., FAST-B contractions); or peak displacement (the highest instantaneous displacement) when the load was not thrown (i.e., CONV, FAST-NB and FAST-B contractions). Peak power, force, and velocity were determined as the highest instantaneous value measured from force onset to force offset (see above). Force onset was defined as the point the force signal increased above the baseline noise envelope during the 300 ms prior to force onset and did not return. Rate of force development (RFD) was measured over the first 100 ms of contraction from force onset. Pilot work indicated that the sled was static throughout this period and thus this initial phase of contraction was isometric. Subsequently assessment of the analysed trials, across all loads and types of contractions, confirmed that sled velocity at 100 ms was < 1% peak velocity and thus was effectively isometric. Therefore, in this initial period, valid RFD measurements and comparisons are possible, without the changes in joint position and velocity that effect on-going force production once movement occurs, and would be expected to confound RFD comparisons between loads and types of contractions (Tillin et al. 2018). Work done during each contraction was calculated by multiplying mean power by movement duration (see above). Finally, to assess the differences in power, force, and velocity throughout the contractions, these variables were measured at specific percentages of time during each of the analysed contractions (10% increments, 0–100% of movement duration). During pilot work, we considered using both displacement- and time-based measurement throughout the contraction. However, displacement-based measurements were skewed to the later phase of each contraction/lift due to the time taken to increase force and overcome the inertia at the start of the lift, with the first 10% of displacement taking 30–40% of the overall movement duration. Time-based increments are also more consistent with the mean values that are averaged over time. Pilot work involving older adults (*n* = 12) assessed the reliability of peak and mean kinetic and kinematic outcomes measured on two separate days (7 days apart) with a load of 50%1RM. The between-session coefficient of variation (CVw; [SD/mean] × 100) was calculated for peak variables (power 3.5%, force 1.7%, and velocity 2.3%), and mean variables (power 5.4%, force 3.1%, and velocity 4.8%).

During isoinertial contractions the RMS amplitude of each sEMG signal was measured over the period of the concentric phase of contraction from displacement onset to force offset/peak displacement (i.e., movement duration). The RMS amplitude of each signal (recording site) was baseline corrected (i.e., resting RMS amplitude was subtracted) and normalised (%) to RMS amplitude at iMVF (knee extensors sites 81%LL; hip extensors and plantar flexors sites 95%LL). The normalised values at each sEMG electrode site were averaged across the two best contractions at each load, before averaging across sites to produce functional muscle group values (KE_EMG_, RF_PROXIMAL_ + RF_DISTAL_ + VL_PROXIMAL_ + VL_DISTAL_ + VM_PROXIMAL_ + VM_DISTAL_ / 6; HE_EMG_, GM + BF + MH/ 3; PF_EMG_, SO + MG + LG / 3). The between-session coefficient of variation (CVw) values were calculated during the above-mentioned pilot work for the functional muscle group sEMG amplitudes (KE_EMG_ 7.7%, HE_EMG_ 17.2%, and PF_EMG_ 28.1%, respectively).

### Statistical analysis

Group data are presented as mean ± SD. Statistical analysis was conducted using SPSS Version 23 (IBM Corp., Armonk, NY) software and the statistical significance was defined as *P* < 0.05. Two factor repeated measures ANOVA was used to assess the effect of contraction type [CONV, FAST-NB and FAST-B] and load [20, 35, 50, 65 and 80%1RM]) on peak and mean measures of force, velocity, power and sEMG amplitude of the functional muscle groups. A further two factor repeated measures ANOVA was used to assess the effect of contraction type [CONV, FAST-NB and FAST-B] and contraction duration [0, 10, 20, …100% of contraction duration] on force, velocity and power, at each load. Where significant main effects were found, a one-way repeated measures ANOVA (CONV vs. FAST-NB vs. FAST-B) was performed at each load or percentage of contraction duration. When a significant one-way ANOVA was found, Bonferroni corrected post-hoc tests were used to make pairwise comparisons between any two types of contractions. Percentage difference (%) was calculated ([[mean^1^ – mean^2^]/mean^1^]*100). Effect sizes (ES; Cohen`s d) were calculated for peak and mean kinetic, kinematic and sEMG values using a pooled standard deviation with ES of < 0.2 “trivial”, ≥ 0.2 to ≤ 0.49 “small”, ≥ 0.5 to ≤ 0.79 “moderate”, and ≥ 0.8 “large”.

## Results

### Contraction descriptors; duration and displacement

A significant main effect for type of contraction was found for both movement duration and displacement, i.e., range of motion (two-way ANOVA; all *P* < 0.001; Table 1.). Contraction duration of CONV was typically similar to the prescribed lifting duration (i.e., 2 s), rising from an average of 1.83 s with the lightest load (20%1RM, and increasing with load to 2.50 s with the heaviest load (80%1RM). Contraction duration was significantly and consistently shorter according to explosive intent FAST-B < FAST-NB < CONV for all loads (one-way ANOVA, all *P* < 0.05). The biggest differences were at the lightest load with FAST-B taking only 19% (0.34 s), and FAST-NB 46% (0.85 s), of the duration of CONV (1.83 s), and the differences in contraction duration became smaller as the load increased. Despite all contractions starting from the same %LL, FAST-B produced greater displacement values for most loads than FAST-NB (50–80%1RM, all *P* < 0.031) and CONV (35–80%1RM, all *P* < 0.003). Whereas FAST-NB and CONV displacements were relatively similar for most loads, and only differed at two loads (20 and 65%1RM, all *P* < 0.05, Table 1). After the initial 100 ms of contraction, from force onset, the period during which RFD was measured, sled velocity was found to be < 1% of peak velocity in all measured contractions confirming that the RFD measurement was effectively isometric (and thus not confounded by changes in joint position or velocity).

**Table 1:** Contraction displacement (m), duration (s) and work (J) during conventional [CONV] vs. fast non-ballistic [FAST-NB] vs. fast ballistic [FAST-B] contractions with each of a range of five loads (20–80%1RM)

LOAD (%1RM)					
	20	35	50	65	80
Displacement (m):					
CONV	0.30 ± 0.03	0.30 ± 0.02	0.30 ± 0.02	0.29 ± 0.02	0.28 ± 0.02
FAST-NB	0.32 ± 0.03^#^	0.32 ± 0.04	0.32 ± 0.03	0.32 ± 0.03^#^	0.30 ± 0.03
FAST-B	0.33 ± 0.03	0.35 ± 0.03^#^	0.36 ± 0.05^#†^	0.35 ± 0.03^#†^	0.35 ± 0.05^#†^
Duration (s):					
CONV	1.83 ± 0.33	2.22 ± 0.45	2.20 ± 0.31	2.40 ± 0.33	2.50 ± 0.34
FAST-NB	0.85 ± 0.20^#^	1.00 ± 0.3^#^	1.10 ± 0.26^#^	1.25 ± 0.31^#^	1.54 ± 0.44^#^
FAST-B	0.34 ± 0.05^#†^	0.47 ± 0.05^#†^	0.63 ± 0.08^#†^	0.85 ± 0.20^#†^	1.32 ± 0.42^#†^
Work (J):					
CONV	48 ± 9	85 ± 15	120 ± 22	155 ± 27	185 ± 29
FAST-NB	51 ± 9	90 ± 17	129 ± 224	165 ± 32	193 ± 42
FAST-B	115 ± 27^#†^	163 ± 39^#†^	190 ± 36^#†^	212 ± 44^#†^	239 ± 46^#†^

Data are presented as the mean + SD

^#^Significantly (P < 0.05) different to CONV

^†^Significantly different to FAST-NB

### Mechanical variables

Contraction type was found to have a main effect on peak and mean measures of power, force, velocity, work and rate of force development (two-way ANOVA; all *P* < 0.002).

### Power

**Fig. 1:**
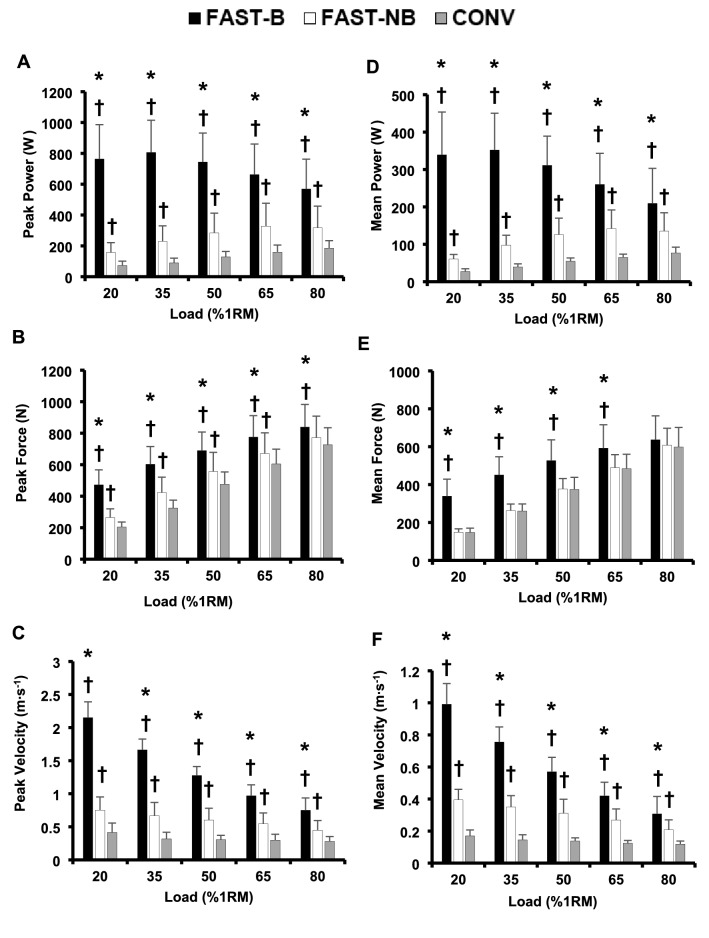
Peak and mean power (**A**, **D**), force (**B**, **E**), and velocity (**C**, **F**) during the concentric phase of conventional [CONV], fast non-ballistic [FAST-NB] and fast ballistic [FAST-B] contractions with a range of five loads (20*–*80%1RM). Data are presented as mean ± SD. *Significantly greater than FAST-NB (*P* < 0.05).^†^Significantly greater than CONV (*P* < 0.05)

There were marked significant differences in mean and peak power between the three types of contractions according to the explosive intent, FAST-B > FAST-NB > CONV, for all loads. Specifically, peak power during FAST-B was 69 to 368% greater than FAST-NB (all *P* < 0.001, ES = 1.4 to 3.7 “Large”), and 221–1033% greater than CONV (all *P* < 0.001, ES = 2.9 to 4.9 “Large”; Fig. 1A) across all loads. Peak power during FAST-NB was also 90–180% higher than CONV with all loads (all *P* ≤ 0.005, ES = 1.6 to 2.2, “Large”, Fig. 1A). Mean power was similarly elevated during FAST-B, being 49 to 466% greater than FAST-NB (all *P* ≤ 0.023, ES = 1.0 to 3.7, “Large”) and 163 to 1172% greater than CONV (all *P* ≤ 0.015; ES = 2.0 to 4.5, “Large”, Fig. 1D) across all loads. FAST-NB also produced greater mean power than CONV with all loads (all *P* ≤ 0.005, + 76 to 147%; ES = 1.6–3.4, “Large”, Fig. 1D).

### Force

Peak force during FAST-B was 10 to 76% greater than FAST-NB (all *P* ≤ 0.005, ES = 0.5 to 2.7, “Moderate to Large”) and FAST-B was 16 to 136% greater than CONV across all loads (all *P* < 0.001, ES = 0.92 to 3.9 “Large”, Fig. 1B). FAST-NB produced 12 to 34% greater peak force than CONV with loads 20–65%1RM (all *P* ≤ 0.007, ES = 0.6 to 1.7, “Moderate to Large”) but not with 80%1RM (*P* = 0.061, Fig. 1B). Mean force was 21 to 132% higher during FAST-B than FAST-NB (all *P* < 0.001, ES = 1.0 to 3.3, “Large”) for loads 20–65%1RM, but not for 80%1RM (*P* = 0.164, Fig. 1E). FAST-B produced 8 to 132% greater mean force than CONV across all loads (all *P* < 0.05, ES = 0.4 to 3.2, “Small to Large”, Fig. 1E). There was no difference in mean force between FAST-NB and CONV during any load (all *P* > 0.54, Fig. 1E).

### Velocity

Considering the full range of loads, peak velocity was 55 to 176% greater during FAST-B than FAST-NB (all *P* < 0.001, ES = 1.8 to 6.7, “Large”) and 178 to 462% higher during FB than CONV (all *P* < 0.001, ES = 3.9 to 11.0, “Large”, Fig. 1C). FAST-NB involved 79 to 133% greater peak velocity than CONV across all loads (all *P* ≤ 0.002, ES = 2.1 to 3.0, “Large”, Fig. 1C). Across all five loads, mean velocity during FAST-B was 147 to 483% greater than CONV (all *P* < 0.001, ES = 2.0 to 4.5, “Large”) and 40 to 150% higher than FAST-NB (all *P* ≤ 0.025, ES = 1.1 to 6.0, “Large”, Fig. 1F). FAST-NB produced 76 to 142% greater mean velocity across all loads than CONV (all *P* ≤ 0.003, ES = 2.0 to 4.2, “Large”, Fig. 1F).

### Work

The work done was markedly greater during FAST-B than both FAST-NB (all *P* ≤ 0.002, + 23 to 125%, ES = 1.0 to 3.1 “Large”) and CONV (all *P* < 0.001, + 29 to 140%, ES = 1.4 to 3.3 “Large”) across all loads measured, but with no differences between FAST-B and CONV (all *P* > 0.054; Table 1). The enhanced work done of FAST-B was most pronounced at the lightest load and smallest at the heaviest load.

### Rate of force development

**Fig. 2:**
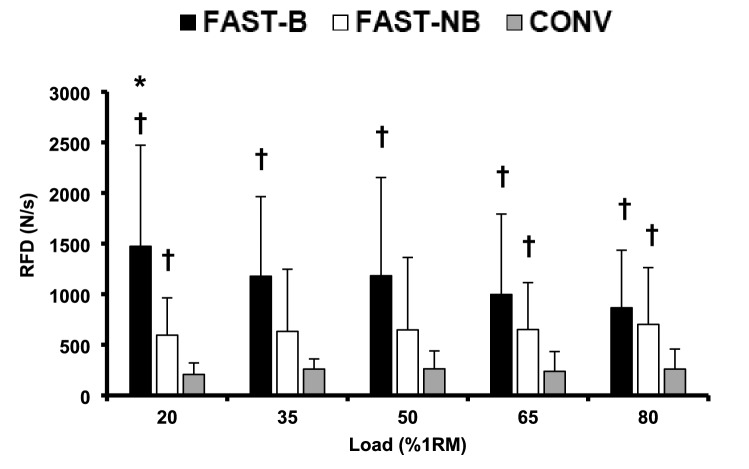
Rate of force development (RFD, 0–100 ms post force onset) for conventional [CONV], fast non-ballistic [FAST-NB] and fast ballistic [FAST-B] contractions with a range of five loads (20*–*80%1RM). Data presented are mean ± SD. *Significantly (*P* < 0.05) greater than FAST-NB. ^†^Significantly (*P* < 0.05) greater than CONV

Considering the full range of loads, FAST-B produced 233 to 612% greater RFD over the first 100 ms of contraction than CONV (all *P* < 0.029, ES = 1.16–1.77 “Large”, Fig. 2). FAST-B produced 148% greater RFD than FAST-NB at the lightest load (P = 0.021, ES = 1.16 “Large”), whereas at moderate loads, despite FAST-B appearing to produce a higher RFD than FAST-NB (35%1RM, + 86%, ES = 0.77 “Large”; 50%1RM, + 83%, ES = 0.63 “Moderate”; 65%1RM, + 53%, ES = 0.53 “Moderate”; 80%1RM, + 23%, ES = 0.29 “Small”) there were no significant differences (P > 0.259). FAST-NB also produced 171 to 190% greater RFD than CONV for most loads (20 and 65–80%1RM, all *P* < 0.042, ES = 1.05–1.42 “Large”), but there were no differences in RFD between FAST-NB and CONV for the remaining two loads (35–50%1RM, all *P* > 0.213, Fig. 2).

### Surface electromyography

**Fig. 3:**
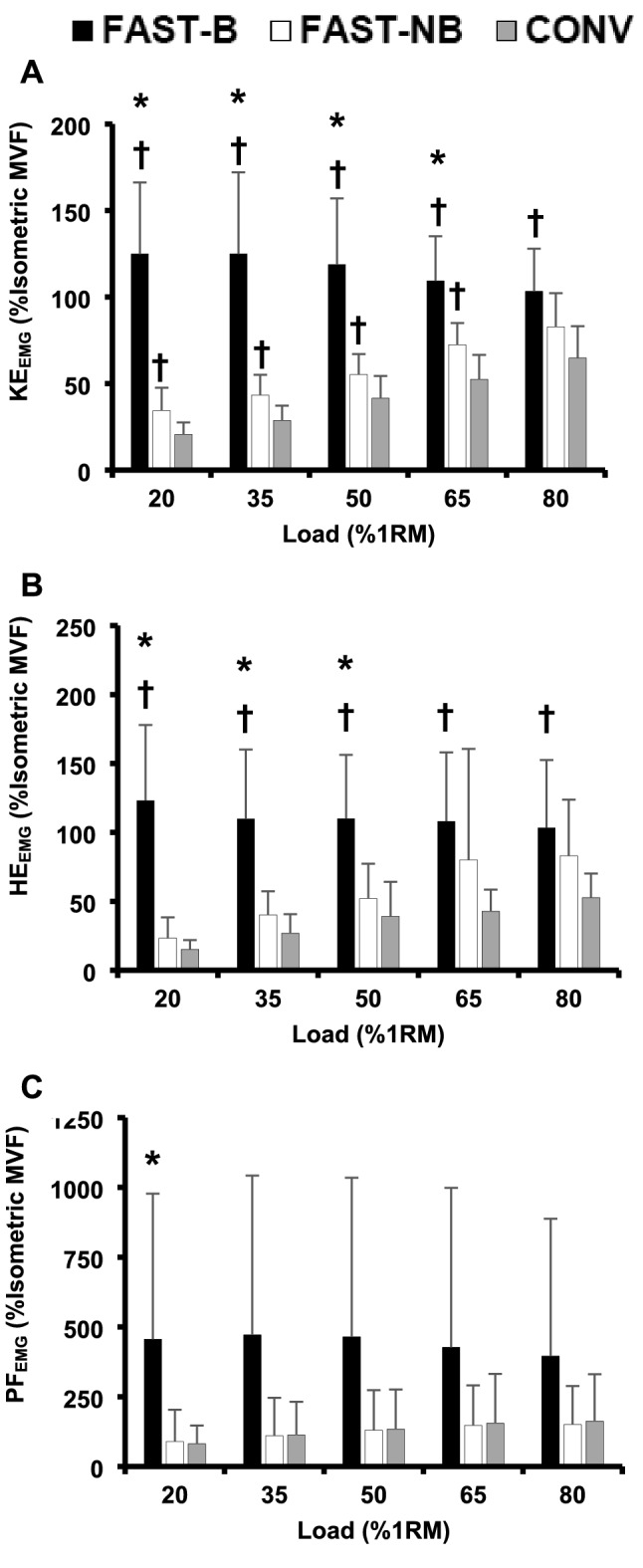
Surface electromyography amplitude (normalised [%] to EMG amplitude at isometric MVF) for the knee extensors (**A**, KE_EMG_), hip extensors (**B**, HE_EMG_), and plantar-flexors (**C**, PF_EMG_) for conventional [CONV], fast non-ballistic [FAST-NB], fast ballistic [FAST-B] contractions with a range of five loads (20*–*80%1RM). Data are presented as mean ± SD. *Significantly (*P* < 0.05) greater than FAST-NB. ^†^Significantly (*P* < 0.05) greater than CONV

Contraction type had a main effect on the normalised RMS amplitude of all three functional muscle groups: KE_EMG_, HE_EMG_, and PF_EMG_ (two-way ANOVA, all *P* < 0.05, Fig. 3A–C). FAST-B contractions involved KE_EMG_ that was 25 to 263% higher than FAST-NB (20–65%1RM; all* P* ≤ 0.005, ES = 0.9 to 3.0, “Large”) and 59 to 505% higher than CONV (20–80%1RM; all *P* ≤ 0.002; ES = 1.8 to 3.5, “Moderate to Large”, Fig. 3A), with the greatest differences at lighter loads (20–50%1RM). FAST-NB produced 39 to 67% higher KE_EMG_ than CONV for most loads (20–65%1RM, all *P* ≤ 0.02; ES = 0.3 to 0.5, “Small to Moderate”, Fig. 3A) with KE_EMG_ becoming similar at the heaviest load. During FAST-B HE_EMG_ was 96 to 712% greater than CONV (20–80%1RM, all *P* ≤ 0.02; ES = 1.4 to 2.8, “Large”) and 111 to 426% greater than FAST-NB (20–50%1RM; all *P* < 0.001; ES = 1.6–2.5, “Large”, Fig. 3B). Whereas FAST-NB involved similar HE_EMG_ to CONV at 4 out of 5 loads. Finally, PF_EMG_ post-hoc analysis revealed that FAST-B was 410% greater than FAST-NB at the lightest load (20%1RM; all *P* ≤ 0.046; ES = 1.0, “Large”, Fig. 3C), but there were no differences between FAST-B and FAST-NB for any other load. There were also no differences between FAST-NB and CON contractions at any load.

### Mechanical variables throughout the contraction

**Fig. 4:**
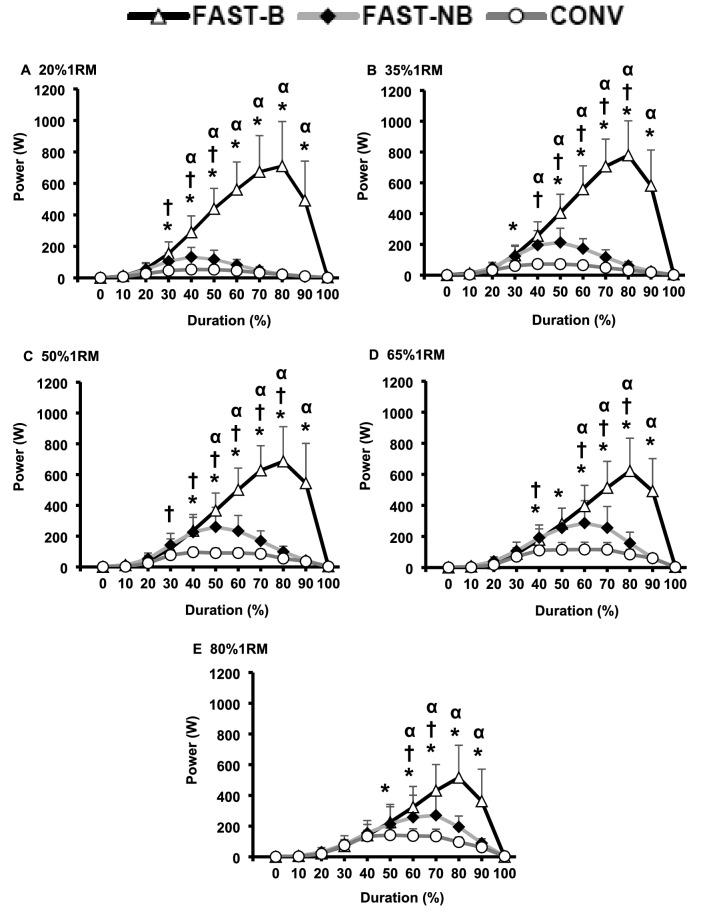
Power production throughout (% movement duration) the concentric phase of contraction for conventional [CONV], fast non-ballistic [FAST-NB], fast ballistic [FAST-B] contractions at each of five different loads (**A**–**E**; 20*–*80%1RM). Data are presented as mean ± SD. Significant differences (*P* < 0.05): *FAST-B vs. CONV; α FAST-B vs. FAST-NB: ^†^FAST-NB vs. CONV

Power, force and velocity measurements showed a time x type of contraction interaction effect for all five loads (two-way ANOVA, All *P* < 0.01). FAST-B produced greater power than both CONV and FAST-NB for an increasing proportion of the contraction as load decreased (differences occurred between the following percentages of contraction duration: 60 to 90% at 80%1RM, up to 40 to 90% at 20%1RM; all *P* < 0.05, Fig. 4A–E). The differences in power between FAST-NB and CONV shifted from relatively early in the contraction with the lightest load (20%1RM, 30 to 50% of contraction duration, all *P* < 0.05), to a longer period during the middle of the contraction with moderate loads (35–50%1RM, 30–80% of contraction duration, all *P* < 0.05) to the later phase of contraction with the heaviest loads (65–80%1RM, 60 to 80% of contraction duration, all *P* < 0.05; Fig. 4).

**Fig. 5:**
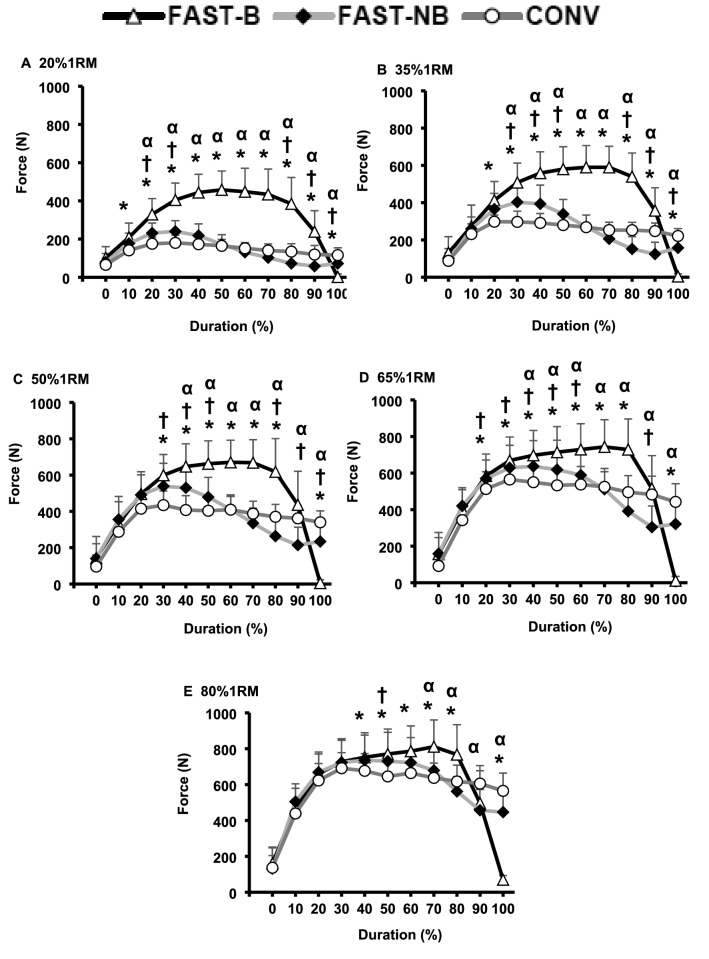
Force production throughout (% movement duration) the concentric phase of contraction for conventional [CONV], fast non-ballistic [FAST-NB], fast ballistic [FAST-B] contractions at each of five different loads (**A**–**E**; 20–80%1RM). Data are presented as mean ± SD. Significant differences (*P* < 0.05): *FAST-B vs. CONV; α FAST-B vs. FAST-NB: ^†^FAST-NB vs. CONV

FAST-B involved greater force production than FAST-NB or CONV throughout most of the contraction duration with light loads (20%1RM, 20 to 90% of contraction duration, all *P* < 0.05, Fig. 5A), but these differences became smaller as load increased, down to only 70 to 80% contraction duration with 80%1RM (e.g., Fig. 5D). With light/moderate loads (20–65%1RM) FAST-NB involved higher force production than CONV during the first part of the contraction, but then lower forces during the latter half of contraction (e.g., Fig. 5C, all *P* < 0.05), although this pattern was not apparent at the highest load (80%1RM, Fig. 4D).

**Fig. 6:**
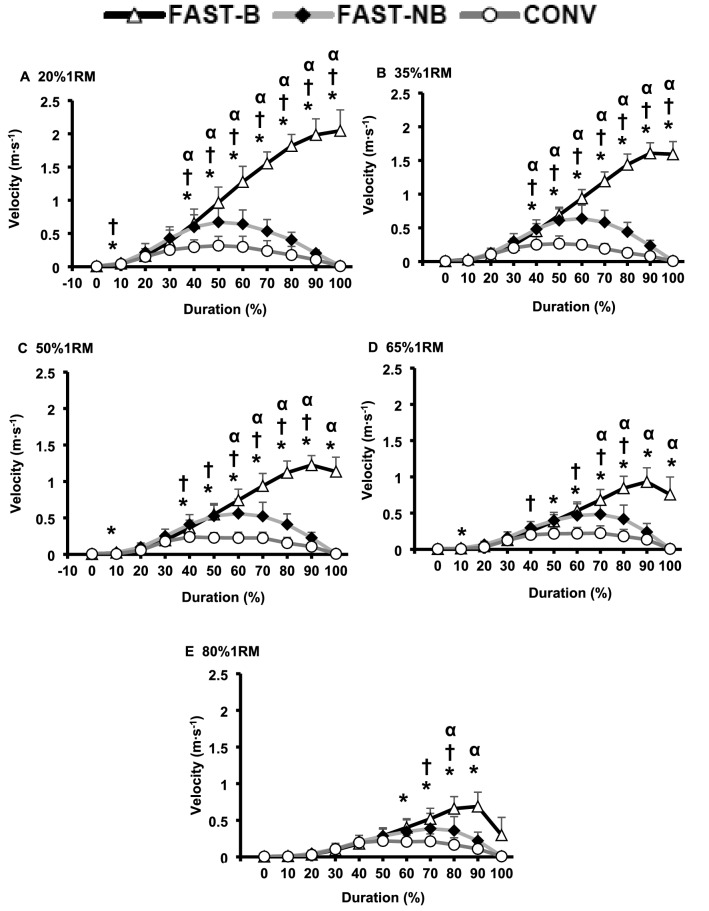
Velocity throughout (% movement duration) the concentric phase of contraction for conventional [CONV], fast non-ballistic [FAST-NB], fast ballistic [FAST-B] contractions at each of five different loads (**A–E**; 20*–*80%1RM). Data are presented as mean ± SD. Significant differences (*P* < 0.05): *FAST-B vs. CONV; α FAST-B vs. FAST-NB:^†^FAST-NB vs. CONV

In general, the velocity values were similar during the initial phase (i.e., 0 to 30% duration) of contraction duration for all three types of contraction. FAST-B contractions produced greater velocities than FAST-NB and CONV for the remainder of the contraction duration with light loads (20–35%1RM, 40 to 100% contraction duration, Fig. 6A–D, all *P* < 0.01), but as the load increased towards the highest loads (e.g., 80%1RM) FAST-B generated higher velocities over a smaller time period than CONV (60 to 90% of contraction duration) or FAST-NB (80 to 90% of contraction duration) contractions (all *P* < 0.05, Fig. 6E). At light loads, FAST-B involved greater velocity for most of the contraction duration (20–35%1RM, 40–90% contraction duration, all *P* < 0.05, Fig. 6A, B) than FAST-NB, but as the load increased these differences became a progressively smaller proportion of the movement (e.g., 80%1RM, only 80 to 90% of contraction duration, all *P* < 0.05, Fig. 6E). Similarly, FAST-NB involved greater velocities than CONV over a large proportion of the contraction at light/moderate loads (20–50%1RM, 40–90% contraction duration, all *P* < 0.05, Fig. 6A–C), but with smaller differences as the load increased (e.g., 80%1RM, 70 to 80% contraction duration; all *P* < 0.05, Fig. 6D–E).

## Discussion

The purpose of this study was to compare the neuromechanics (power, force, velocity and sEMG) of different types of leg press isoinertial contractions (FAST-B vs. FAST-NB vs. CONV) across a range of RT loads in older adults to inform the development of RT programmes. The main finding was that mean and peak power were markedly different between the three types of contractions according to the explosive intent, FAST-B > FAST-NB > CONV, for all loads. The findings, therefore, support the hypothesis that FAST-B contractions generate greater peak and mean neuromuscular power than both FAST-NB (+ 49–466%) and CONV (+ 163–1172%) contractions irrespective of the load lifted. The greater mean and peak power performance during FAST-B was due to a combination of both greater force (+ 10–136%) and velocity (+ 40–483%) than FAST-NB and CONV, with the greater mechanical outputs underpinned by higher KE_EMG_ and HE_EMG_ for most loads. This current study highlights that fast/explosive RT contractions (i.e., both FAST-B and FAST-NB) generated substantially greater neuromuscular power than widely recommended CONV contractions in older adults. Interestingly FAST-B contractions also produced far superior power performance than FAST-NB contractions, and therefore, ballistic intent during the concentric phase of contraction appears essential for optimal power production. These findings indicate that FAST-B contractions may provide a substantially superior stimulus for chronic power adaptation than both CONV or FAST-NB contractions.

The current study has for the first time documented the neuromechanics of different fast/explosive contractions while also comparing these contractions to the widely recommended slow controlled CONV RT contractions (ACSM guidelines, Ratamess et al. 2009) across a range of loads in the lower limb which has great functional significance for older adults. Given that muscle power is the index of neuromuscular function most sensitive to ageing (Skelton et al. 1994) and a critical determinant of both fall risk (Perry et al. 2007) and functional independence (Foldvari et al. 2000) amongst older adults, it appears important to optimise RT for the development/maintenance of muscle power in older adults. Investigation of the acute neuromechanics of resistance exercise appears a key step in highlighting contractions/regimes that merit further investigation in longitudinal intervention studies. While FAST-NB RT has begun to receive some scientific attention (Ratamess et al. 2009; Fragala et al. 2019) as it requires deceleration during the contraction, power production is inevitably constrained (Fragala et al. 2019). Whereas FAST-B RT has been shown in young adults to provide a potentially greater power stimulus than FAST-NB RT (Newton et al. 1996; Cronin and Marshall, 2003), but with no evidence in older adults.

The major finding of this study was that neuromuscular power, assessed by both mean and peak, was consistently different between the types of contraction, specifically FAST-B > FAST-NB > CONV. Interestingly, while power production during FAST-NB also exceeded CONV, FAST-NB contractions were less than half of the difference between CONV and FAST-B contractions (i.e., closer to CONV than FAST-B) for both mean and peak power with all loads, indicating that FAST-B contractions produced particularly pronounced power output. Thus, moving with fast/explosive intent in the first part of the movement does enhance power production in comparison to slower more controlled lifts, but the intent to move fast throughout the whole lift and attempting to ballistically throw the load produces an additional even larger enhancement in power. The distinctiveness of FAST-B contractions was reinforced as they generated greater mechanical work than both CONV and FAST-NB, with no benefit of FAST-NB vs. CONV. The higher mean power of FAST-B in the current study extends some previous research in younger adults during bench press exercise that found greater mean power across a range of loads (15–75%1RM; + 10–60% (Frost et al. 2008) and greater mean and peak power for a single load (i.e., 45%1RM; + 67–70%, Newton et al. 1996) during FAST-B than FAST-NB contractions. Although another study utilising only a single load (45%1RM) found no differences in mean power between FAST-B and FAST-NB during squat exercise in young men (Lake et al. 2012) The discrepancies reported for power output may be explained by methodological differences, including: the study population (age and resistance training experience), the specific exercise performed (bench press vs. leg press), the occurrence of a prior eccentric lowering phase (i.e., before the measured concentric phase) and finally whether participants had to control the projected load.

The effect of type of contraction on power performance (i.e., FAST-B > FAST-NB > CONV) indicates that the type of contraction may influence the improvements in power in response to chronic RT. Previous research in older adults has found that chronic exposure to FAST-NB RT (i.e., a training program) is better than CONV RT for improving older adult muscle power (Sayers and Gibson, 2012; Bottaro et al. 2007; Straight et al. 2016). However, given the superiority of FAST-B during single repetitions than FAST-NB, with greater power and more work done it would be reasonable to assume that FAST-B RT would provide a more potent stimulus for power adaptation than FAST-NB and CONV RT in older adults. However, the chronic power adaptations to FAST-B RT remains to be explored in any population and warrants investigation. In addition to the explosive intent, while not an explicit aim of the current study, the findings further reinforced that the load used may also affect power production (Kraemer et al. 2002; Ratamess et al. 2009). Power RT guidelines typically prescribe a broad loading range for optimising power production (i.e., 30–60%1RM, Ratamess et al. 2009; Fragala et al. 2019). Interestingly, the current study found the shape of the load–power relationship was also dependent of the explosive intent, with FAST-B and FAST-NB contractions producing their highest power values at loads of 35% and 65%1RM, respectively, with the latter being outside of the range of loads prescribed for power RT.

When comparing fast/explosive contractions (FAST-B and FAST-NB) to CONV, mean power was clearly greater with fast/explosive contractions and this was due to a combination of elevated mean force and mean velocity during the fast/explosive contractions. Thus, the intent to move fast appears imperative for enhancing power production compared to CONV slow controlled contractions. The fast/explosive contractions generally had a steeper rise in force (i.e., higher rate of force development) during the isometric phase of contraction before movement occurred and had higher forces during the first half of the concentric contraction. These higher forces appeared to precede higher movement velocities during the mid-and/or late phase of fast-type contractions leading to elevated mean velocity and a shorter movement duration, and consequently also higher mean power production. When contrasting the fast/explosive contractions, FAST-B and FAST-NB produced relatively similar power, force and velocity during the early phase of movement duration (0–30% of movement duration). During the initial isometric phase of the contraction, RFD only differed between FAST-B and FAST-NB at the lightest load. At moderate loads RFD appeared to be somewhat higher for FAST-B vs. FAST-NB (35–65%1RM, + 23–86%), but these differences were non-significant likely due to extensive between participant variability. Thus, during both the isometric and early dynamic phase of contraction (up to 30% of movement duration) FAST-B and FAST-NB were relatively similar, likely, because for both these types of contractions, the instruction was to move “as fast as possible” during the first part of the movement. These findings might imply that for training purely these early explosive phases of contraction that FAST-B and FAST-NB are relatively similar. However, as the contractions continued, force and velocity, and thus also power, were compromised during the FAST-NB contraction (vs. FAST-B). This can be clearly seen in the force values during FAST-NB contractions which were relatively similar to FAST-B during the early phase of contraction but then declined (for FAST-NB), even to below that of CONV during the second half of the contraction. This decline in force with FAST-NB appears to have been necessitated by the need to limit velocity, which also declined markedly throughout the second half of the FAST-NB contractions, to complete the contraction with the prescribed velocity of 0 at the end of the movement (i.e., no projection of the load). Therefore, the lower force and thus also velocity and power values of FAST-NB compared to FAST-B contractions appear to be a direct consequence of the need to decelerate and avoid projecting the load. The current findings, therefore, support the hypothesis that FAST-B contractions produce superior mean power, force and velocity due to the greater time spent accelerating the load throughout the whole range of motion (Frost et al., 2010).

This study was the first to comprehensively assess older adult lower limb sEMG amplitude during different types of concentric isoinertial contractions (FAST-B vs. FAST-NB vs. CONV) and across a range of RT loads. Overall, it was found that FAST-B contractions produced greater KE_EMG_ than both CONV and FAST-NB with all loads, and higher and HE_EMG_ during FAST-B than CONV at all loads and FAST-NB for most loads (i.e., 20–50%1RM). Thus, supporting a previous study that found FAST-B contractions involved greater sEMG amplitudes than FAST-NB contractions (Frost et al. 2008). However, the Frost et al. (2008) study only compared FAST-B and FAST-NB contractions in a young athletic population. It is also important to consider that the substantially greater KE_EMG_ and HE_EMG_ during FAST-B than CONV could be partially confounded by the differences in movement velocity which may produce movement artefacts and also alter the location of the electrodes relative to the underlying muscle fibres as the muscles shorten and contract (De Luca, 1997). Nonetheless the substantially greater KE_EMG_ and HE_EMG_ with FAST-B is consistent with the greater force production, and thus also velocity and power, that appear to be a consequence of greater sEMG amplitudes. PF_EMG_ was not greater during FAST-B than FAST-NB and CONV contractions, which appears to explain the high variability of PF_EMG_ during FAST-B contractions (see the error bars within Fig. 3C). Finally, a limitation of this current study was the normalisation of the sEMG amplitudes during the isoinertial contractions to sEMG amplitudes during isometric contractions at two positions (i.e., 81 and 95%LL). Whereas a more robust protocol would have established a sEMG-displacement relationship and thus made sEMG normalisation position specific through the range of movement (i.e., 74–100%LL).

## Practical and future applications

FAST-B contractions performed with standard isoinertial (mass loaded) equipment involves the purposeful ballistic projection of the load by the participant, which provides a practical challenge of safely managing the projected load. Currently commercially available isoinertial RT equipment have not typically been designed for the safe projection of loads, in part perhaps because the evidence base for RT with FAST-B contractions has not been convincing. If the evidence base supporting the use of FAST-B RT increases, and the technology of isoinertial resistance exercise machines improves, this facility may become more widely available. Alternatively, other forms of RT machines, i.e., pneumatic or electrically braked, may facilitate safe maximal movement velocity contractions (i.e., fast/explosive intent) without having a projected load. However, this apparatus is also not widely available and while there are some promising findings (Bottaro et al. 2007; Sayers and Gibson, 2012; Balachandran et al. 2017), both the acute neuromechanics and physiological adaptations to RT with these types of resistance have had relatively limited scientific attention. Therefore, until safe FAST-B enabled RT machines become more accessible, older adults looking to maintain/improve muscle power with isoinertial equipment should perform FAST-NB rather than CONV contractions. Moreover, there is a need to better understand the value of FAST-B contractions in older adults and future research should investigate the long-term training adaptations (i.e., power performance, muscle size, neuromuscular activation etc.) to this type of contraction to better prescribe training interventions for older adults.

## Conclusions

In conclusion, neuromuscular power was consistently different between the types of contraction, specifically FAST-B > FAST-NB > CONV, such that FAST-B contractions produced markedly more power than both CONV slow, but also FAST-NB, contractions irrespective of the load. Furthermore, the greater power performance during FAST-B contractions was facilitated by greater force and velocity performance which was related to higher muscle activation during FAST-B than both CONV and FAST-NB. Due to the substantially greater power production during FAST-B contractions, this type of contraction may provide a markedly greater stimulus for power development during a RT programme.

## Supplementary Information

Below is the link to the electronic supplementary material.

**Supplementary file 1: Digital Content 1.** The linear leg press dynamometer used to measure isometric and isoinertial muscle function. The dynamometer was an inclined (30°) linear leg press that consisted of a sled that could be fixed in position (i.e. percentage of leg length) for isometric measurements and the sled could also move dynamically along the leg press runners. (**A**) A force plate was mounted to the sled perpendicular to the direction of travel and the draw-wire (**B**) displacement transducer housing was anchored to the base of the frame, with the extendable draw-wire attached to the moveable sled.

## Abbreviations

BF: Biceps femoris
BMI: Body mass index
CONV: Conventional
CV_W_: Coefficient of variation
ES: Effect size
FAST-B: Fast ballistic
FAST-NB: Fast non-ballistic
GM: Gluteus maximus
IPAQ: International physical activity questionnaire
LG: Lateral gastrocnemius
MG: Medial gastrocnemius
MH: Medial hamstring
MVC: Maximum voluntary contraction
%LL: Percentage of leg length
%1RM: Percentage one repetition maximum
RF: Rectus femoris
RT: Resistance training
RMS: Root mean square
sEMG: Surface electromyography
SO: Soleus
VL: Vastus lateralis
VM: Vastus medialis

## References

[CR1] Accettura AJ, Brenneman EC, Stratford PW, Maly MR (2015). Knee extensor power relates to mobility performance in people with knee osteoarthritis: cross-sectional analysis. Phys Ther.

[CR2] Balachandran AT, Gandia K, Jacobs KA, Streiner DL, Eltoukhy M, Signorile JF (2017). Power training using pneumatic machines vs. plate-loaded machines to improve muscle power in older adults. Exp Gerontol.

[CR28] Bassey EJ, Fiatarone MA, O'Neill EF, Kelly M, Evans WJ, Lipsitz LA. (1979) Leg extensor power and functional performance in very old men and women. Clinical science. 82(3):321–327. 10.1042/cs0820321 1312417

[CR3] Berger MJ, McKenzie CA, Chess DG, Goela A, Doherty TJ (2012). Quadriceps neuromuscular function and self-reported functional ability in knee osteoarthritis. J Appl Physiol.

[CR4] Buatois S, Perret-Guillaume C, Gueguen R (2010). A simple clinical scale to stratify risk of recurrent falls in communitydwelling adults aged 65 years and older. Phys Ther.

[CR5] Craig CL, Marshall AL, Sjöström M (2003). International physical activity questionnaire: 12-country reliability and validity. Med Sci Sports Exerc.

[CR6] Cronin J, Marshall RN (2003). Force-velocity analysis of strength-training techniques and load: implications for training strategy and research. J Strength Cond Res.

[CR7] Davies DSC, Atherton F, McBride M, Calderwood C (2019) UK Chief Medical Officers’ Physical Activity Guidelines.” Department of Health and Social Care, no. September: 1–65. https://www.gov.uk/government/publications/physical-activity-guidelines-uk-chief-medical-officers-report. Accessed: 08 Mar 2020.

[CR8] De Luca CJ (1997). The use of surface electromyography in biomechanics. J Appl Biomech.

[CR9] Foldvari M, Clark M, Laviolette LC (2000). Association of muscle power with functional status in community-dwelling elderly women. J Gerontol A Biol Sci Med Sci.

[CR10] Folstein MF, Folstein SE, McHugh PR (1975). ‘Mini-Mental State’. A practical method for grading the cognitive state of patients for the clinician. J Psychiatr Res.

[CR11] Fragala MS, Cadore EL, Dorgo S, Izquierdo M, Kraemer WJ, Peterson MD, Ryan ED (2019). Resistance training for older adults: position statement from the national strength and conditioning association. J Strength Cond Res.

[CR12] Frost DM, Cronin JB, Newton RU (2008). A comparison of the kinematics, kinetics and muscle activity between pneumatic and free weight resistance. Eur J Appl Physiol.

[CR13] Frost DM, Cronin J, Newton RU (2010). A biomechanical evaluation of resistance: fundamental concepts for training and sports performance. Sports Med.

[CR14] Izquierdo M, Merchant RA, Morley JE (2021). International Exercise Recommendations in Older Adults (ICFSR): expert consensus guidelines. J Nutr Heal Aging.

[CR15] Kraemer WJ, Adams K, Cafarelli E (2002). Progression models in resistance training for healthy adults. Med Sci Sports Exerc.

[CR16] Lake J, Lauder M, Smith N, Shorter K (2012). A comparison of ballistic and nonballistic lower-body resistance exercise and the methods used to identify their positive lifting phases. J Appl Biomech.

[CR17] Murray AM, Thomas AC, Armstrong CW, Pietrosimone BG, Tevald MA (2015). The associations between quadriceps muscle strength, power, and knee joint mechanics in knee osteoarthritis: a cross-sectional study. Clin Biomech.

[CR18] Newton RU, Kraemer WJ, Hakkinen K, Humphries BJ, Murphy AJ (1996). Kinematics, kinetics, and muscle activation during explosive upper body movements. J Appl Biomech.

[CR19] Perry MC, Carville SF, Smith ICH, Rutherford OM, Newham DJ (2007). Strength, power output and symmetry of leg muscles: effect of age and history of falling. Eur J Appl Physiol.

[CR20] Piercy KL, Troiano RP, Ballard RM, Carlson SA, Fulton JE, Galuska DA, George SM, Olson RD (2018). The Physical Activity Guidelines for Americans. JAMA.

[CR21] Ratamess NA, Alvar BA, Evetoch TK (2009). Progression models in resistance training for healthy adults. Med Sci Sport Exerc.

[CR22] Reid KF, Martin KI, Doros G (2015). Comparative effects of light or heavy resistance power training for improving lower extremity power and physical performance in mobility-limited older adults. J Gerontol A Biol Sci Med Sci.

[CR23] Rodriguez-Lopez C, Alcazar J, Sánchez-Martín C, Ara I, Csapo R, Alegre LM (2020). Mechanical characteristics of heavy vs. light load ballistic resistance training in older adults. J Strength Cond Res.

[CR24] Sayers SP, Gibson K (2012). Effects of high-speed power training on muscle performance and braking speed in older adults. J Aging Res.

[CR25] Skelton DA, Greig CA, Davies JM, Young A (1994). Strength, power and related functional ability of healthy people aged 65–89 years. Age Ageing.

[CR26] Straight CR, Lindheimer JB, Brady AO, Dishman RK, Evans EM (2016). Effects of resistance training on lower-extremity muscle power in middle-aged and older adults: a systematic review and meta-analysis of randomized controlled trials. Sport Med.

[CR27] Tillin NA, Pain MT, Folland JP (2018). Contraction speed and type influences rapid utilisation of available muscle force: neural and contractile mechanisms. J Exp Biol.

