# Supplementary figures and images for: Fast and ballistic contractions involve greater neuromuscular power production in older adults during resistance exercise
Source: Eur J Appl Physiol. 2022 Apr 16;122(7):1639–55. doi: 10.1007/s00421-022-04947-x (PMC9197913; doi:10.1007/s00421-022-04947-x)

**Supplemental digital content**


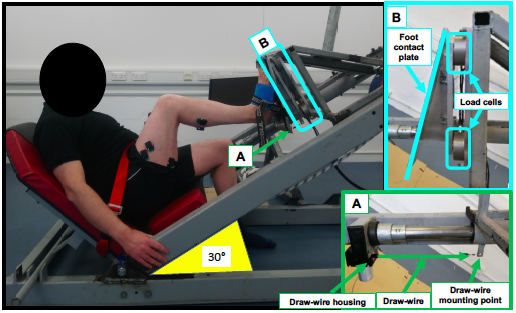

Supplement: Supplementary file 1 — Digital Content 1. The linear leg press dynamometer used to measure isometric and isoinertial muscle function. The dynamometer was an inclined (30°) linear leg press that consisted of a sled that could be fixed in position (i.e. percentage of leg length) for isometric measurements and the sled could also move dynamically along the leg press runners. (A) A force plate was mounted to the sled perpendicular to the direction of travel and the draw-wire (B) displacement transducer housing was anchored to the base of the frame, with the extendable draw-wire attached to the moveable sled. [file 421_2022_4947_MOESM1_ESM.docx]
